# The Effect of Tissue-Mimicking Phantom Compressibility on Magnetic Hyperthermia

**DOI:** 10.3390/nano9050803

**Published:** 2019-05-25

**Authors:** Katarzyna Kaczmarek, Radosław Mrówczyński, Tomasz Hornowski, Rafał Bielas, Arkadiusz Józefczak

**Affiliations:** 1 Institute of Acoustics, Faculty of Physics, Adam Mickiewicz University, Uniwersytetu Poznańskiego 2, 61-614 Poznań, Poland; katarzyna.kaczmarek@amu.edu.pl (K.K.); hornaku@amu.edu.pl (T.H.); rafal.bielas@amu.edu.pl (R.B.); 2 NanoBioMedical Centre, Adam Mickiewicz University, Wszechnicy Piastowskiej 3, 61-614 Poznań, Poland; radoslaw.mrowczynski@amu.edu.pl

**Keywords:** magnetic nanoparticles, magnetic hyperthermia, Brown relaxation, compressibility, tissue-mimicking phantoms

## Abstract

During hyperthermia, magnetite nanoparticles placed in an AC magnetic field become a source of heat. It has been shown that in fluid suspensions, magnetic particles move freely and generate heat easily. However, in tissues of different mechanical properties, nanoparticle movement is limited and leads to a small temperature rise in tissue. Therefore, it is crucial to conduct magnetic hyperthermia experiments in similar conditions to the human body. The effect of tissue-mimicking phantom compressibility on the effectiveness of magnetic hyperthermia was investigated on agar phantoms. Single and cluster nanoparticles were synthesized and used as magnetic materials. The prepared magnetic materials were characterized by transmission electron microscopy (TEM), and zeta potential measurements. Results show that tissue-mimicking phantom compressibility decreases with the concentration of agar. Moreover, the lower the compressibility, the lower the thermal effect of magnetic hyperthermia. Specific absorption rate (SAR) values also proved our assumption that tissue-mimicking phantom compressibility affects magnetic losses in the alternating magnetic field (AMF).

## 1. Introduction

Iron oxide nanoparticles in tumor tissues can act as a heat source because of their intrinsic magnetic features and nanosize. Once tissue with embedded nanoparticles is placed in an alternating magnetic field, the energy from the magnetic field is transformed to heat. Heat transferred from the nanoparticles to the tissue increases its temperature. In the case of superparamagnetic particles, the major heating mechanism is relaxation. The magnetic moment of magnetic nanoparticles tends to align with the direction of the magnetic field through the movement of the magnetic moment within a nanoparticle (Néel relaxation) or the rotation of the whole nanoparticle (Brown relaxation) [1,2,3,4,5]. Very high specific absorption rate (SAR) values of nanoparticle-based magnetic hyperthermia have been obtained for many years. Most research focuses on the development (synthesis and production) of new types of nanoparticles with different shapes, grain and hydrodynamic sizes, magnetic properties, and biocompatible surfactant layers. However, the parameters of nanoparticles that are mentioned are not the only factors influencing the effectiveness of hyperthermia. The heating effect also strongly depends on the medium in which the nanoparticles have to act. It has been shown that magnetic heating works very well in aqueous suspensions [1,2,5]. However, high heating power in aqueous suspensions may not translate into efficient heating in a cellular environment [6]. In magnetic fluids, particles move freely, whereas in tissue their movement may be limited due to interaction with cells, which alters the particle magnetic relaxation. Various tissues and tumors have different mechanical properties that change as the tumor grows [7]. Solid tumors during growth undergo stiffening and, in general, exhibit higher stiffness and lower elastic moduli than their host’s normal tissues [8,9,10]. Mechanical properties play a crucial role in the growth, development, and therapeutics of tumors [9]. Tumor rigidity may compromise the efficacy of magnetic hyperthermia. Therefore, it is important to understand the influence of mechanical properties on magnetic thermal therapy. Magnetic hyperthermia experiments with nanoparticles should be performed in materials with different elastic properties. To test this phenomenon, tissue-mimicking phantoms (hydrogel) may be used. Hydrogels are commonly used in biomedical applications because of their physical properties, which are similar to those of living tissue.

Various phantoms suitable for hyperthermia studies have been produced in many laboratories over the last decade [11,12,13]. Agar-based tissue-mimicking materials are specially developed systems that mimic the physical properties of various human tissues. They have been invaluable for developing and testing the thermal effects of hyperthermia [14]. Agar-based thermo-reversible gels consist of thick bundles of agar chains linked by hydrogen bonds, with pores holding water. The mechanical properties of agar-gel to a large extent depend on the mesh-size network (pore size), which is related to agar concentration. With an increase in agar concentration, the pore size decreases due to an increased rate of nucleation and closer packing of the chains. The pore size increases with the setting temperature due to melting of the weak junctions [15]. Because a higher agar concentration produces noticeably stiffer materials, it is reasonable to expect that in phantoms it will correspond to a higher bulk modulus. Stiffness differences result primarily from differences in dry-weight agar concentration [16]. 

The mechanical properties of phantoms may be evaluated by an acoustic technique that allows one to measure the compressibility of the phantom. In general, ultrasound velocity depends on the bulk modulus *K* and shear modulus *G* and can be expressed as *c* = ((*K* + $\frac{4}{3}$*G*)/*ρ*)^1/2^, where *ρ* is the density of a medium. For fluids, the shear modulus *G* equals zero. For agar phantoms consisting mainly of water, the shear modulus *G* is six orders of magnitude smaller than *K* [17]; therefore, we considered it negligible for our study. When the shear modulus is negligible, we can use a sound velocity equation in the form *c* = (*ρβ_s_*)^−1/2^ to estimate adiabatic compressibility *β_s_*. Adiabatic compressibility is an inverse of the bulk modulus *K*. Therefore, ultrasound velocity measurements offer a favorable method for determining the compressibility and bulk modulus of phantoms.

In this study, we analyzed how mechanical properties, such as compressibility, affect magnetic losses in the alternating magnetic field. Magnetic nanoparticles and colloidal clusters composed of multiple magnetic nanoparticles have shown great potential in hyperthermia heating, so single and cluster nanoparticles were synthesized and used as magnetic materials. The effect was examined in particle suspension (magnetic fluid) and ferrogel with various values of compressibility. We demonstrated that an environment in which nanoparticles are embedded affects the temperature increase during magnetic hyperthermia.

## 2. Materials and Methods 

### 2.1. Magnetic Particles

Two types of magnetic materials were synthetized to be utilized in our experiments, single nanoparticles (SNPs) and nanosphere cluster nanoparticles (CNPs) composed of small magnetite nanograins.

The reagents used in this work, purchased from Sigma-Aldrich (Hong Kong, China), were iron(III) chloride hexahydrate, iron(II) chloride tetrahydrate, ammonium solution (25%), citric acid, sodium acetate, sodium acrylate, ethylene glycol, and diethylene glycol.

In order to obtain SNPs, FeCl_2_·4H_2_O (1.7 g, 8.57 mmol) and FeCl_3_·6H_2_O (4.7 g, 16.2 mmol) were dissolved in 80 mL of deionized water [18]. Then, the solution was degassed with nitrogen and heated up to 90 °C, followed by the addition of 20 mL of 25% ammonia aqua. The heating continued for 30 min, and then 8 mL of 20% citric acid was added. The mixture was kept for 1.5 h at 90 °C. Finally, the magnetic nanoparticles were collected by an external magnetic field and rinsed with deionized water.

The CNPs were obtained according to Xuan et al. [19]. Briefly, sodium acrylate (1.5 g, 16 mmol), sodium acetate (1.5 g, 18.3 mmol) and FeCl_3_·6H_2_O (0.54 g, 2 mmol) were stirred in the mixture of ethylene glycol and diethylene glycol until all compounds dissolved. Next, the mixture was transferred to the Teflon autoclave and kept at 200 °C for 10 h. The mixture was cooled down and particles were collected by centrifugation and washed with EtOH. The obtained material was dried in a vacuum. Finally, the particles were redispersed in water at a concentration of 1 mg/mL.

**Figure 1:**
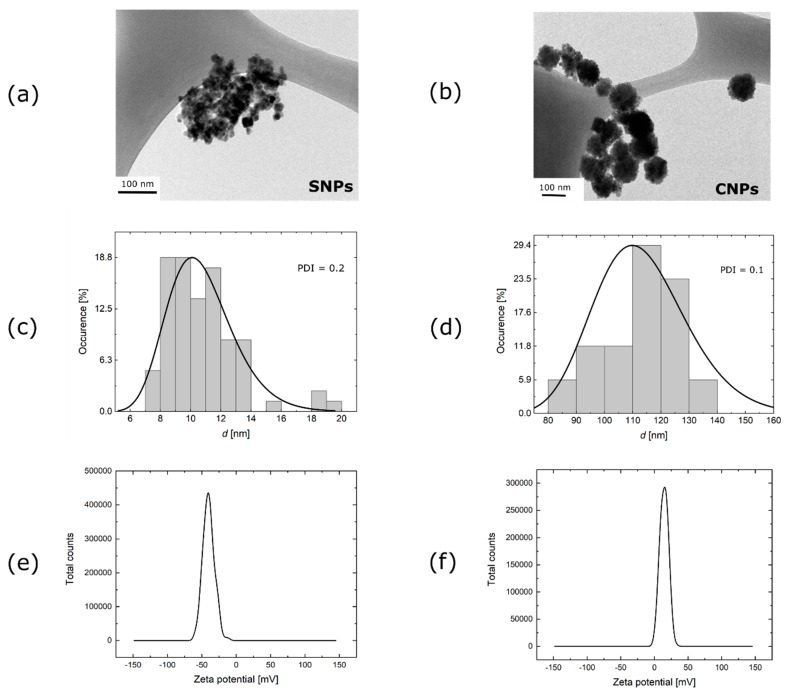
The characterization of nanoparticles: transmission electron microscopy (TEM) micrograph for (**a**) single nanoparticles (SNPs), (**b**) cluster nanoparticles (CNPs). Particle size distribution from TEM with calculated polydispersity index (PDI) for (**c**) SNPs and (**d**) CNPs. Zeta potential of (**e**) SNP water suspension and (**f**) CNP water suspension.

Morphology of the SNPs and CNPs was determined by transmission electron microscopy (TEM). The TEM images were recorded on a JEM-1400 microscope made by JEOL-1400 (Tokyo, Japan) with an accelerating voltage of 120 kV. Samples were dropped cast on a copper grid (Formvar/Carbon 200 mesh made by TedPella (USA)) after 5–15 min of sonication and dried in a vacuum desiccator. For SNPs, TEM revealed a spherical shape of between 8 and 14 nm (Figure 1a). Zeta potential measurements were determined by Zetasizer Nano ZS made by Malvern Instruments Ltd. (Malvern, United Kingdom).

The electric charges on a nanoparticle surface play an important role in the physical stability of nanoparticle-based suspensions. Nanoparticles with zeta potentials values greater than +30 mV or more negative than −30 mV repel each other and show no tendency to aggregate, thus affording high stability in water [20]. Moreover, the nanoparticle tendency to agglomerate, dependent on surface charges, may affect the hyperthermic effect. We concluded that the obtained SNPs exhibit a strongly negative charge, i.e., −40 mV, which is supposed to assure the colloidal stability of the medium. Single nanoparticles were more stable than CNPs; however, they still had a small tendency to aggregate. 

To characterize CNPs, the same techniques were used. The TEM images showed that CNP sizes were between 110 and 130 nm (Figure 1b). The zeta potential of CNPs showed a slightly positive charge of around +16 mV (Figure 1f). A slightly positive charge was responsible for their lower stability compared to SNPs. 

### 2.2. Tissue-Mimicking Phantoms

In our study, all experiments were performed on agar-based phantoms. Agar consists of a mixture of agarose (the predominant component) and agaropectin [21]. The gelation mechanism is explained as a nucleation and growth process [15]. Three stages can be identified: induction, gelation, and pseudo-equilibrium. Upon cooling down, liquid–liquid phase separation occurs with the formation of nuclei composed of polymer-rich phases. During this induction stage, the sample remains in the sol state. As gelation progresses, the nuclei grow and form a network of polymer-rich phases. The polymer-rich phases tend to coagulate so as to minimize the interface between the polymer-rich and polymer-poor phases, thereby reducing the interfacial free energy. The rigidity of the agarose chains and the aggregation of the chains within the polymer-rich phase prevents large-scale coagulation. Local merging of the polymer-rich phases leads to continuously increasing pore size. In the later part of the gelation stage, most of the agarose chains are immobilized in the fibrous junctions, and local coagulation becomes more difficult [15].

For our experiments, we prepared agar phantoms doped with SNPs or CNPs. The weight concentration of the agar in the phantoms was 3% and 7% (*w/w*), whereas the concentration of the magnetic nanomaterial was 1% (*w/w*). The compressibilities *β_s_* for 3% and 7% agar phantom were 4.925 × 10^−10^ m^2^/N and 4.491 × 10^−10^ m^2^/N. The phantom had a cylindrical shape with a diameter of 3 cm and a height of approximately 2.5 cm. The employed agar powder is classified by the company Sigma-Aldrich as a plate count agar (product number: 88588).

### 2.3. Measurement Setup

#### 2.3.1. Ultrasonic Wave Velocity Measurements

**Figure 2:**
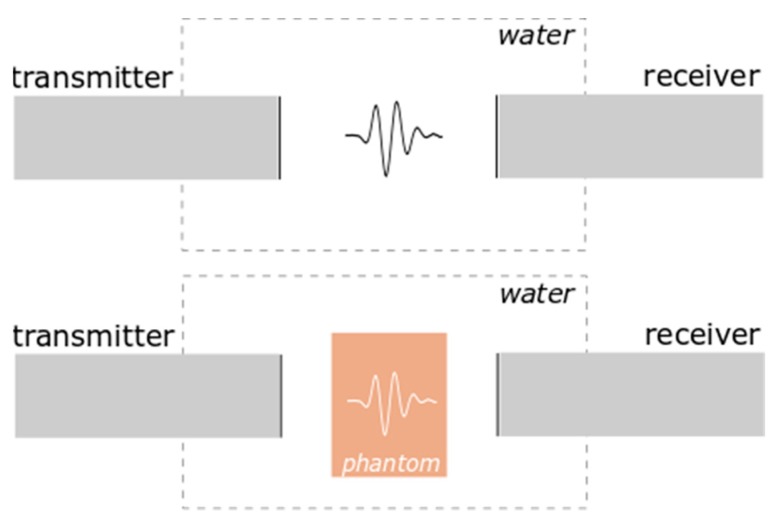
Experimental setup for ultrasonic wave velocity measurements.

To measure the acoustic velocity, an ultrasonic insertion technique [22] was used. This technique is a relative measurement method of the transmission of longitudinal ultrasonic waves through gel embedded in an aqueous environment [22]. It is based on the measurement of a transit time of a pulse between a pair of transmit and receive transducers. The experimental setup is presented in Figure 2. The transducer operated in transmission mode and was driven by an Optel Pulser/Receiver Card 01/100, which provided a unipolar spike pulse with an amplitude of 360 V and a fall time better than 20 ns. The received signal was sampled at a rate of 100 MS/s and recorded. The ultrasonic velocities in the samples were determined from the temporal shift Δ*t = t_w_* − *t_p_* between the pulse transit times *t_w_* without the phantom and *t_p_* with the phantom [23]. The velocity of an ultrasonic wave can be calculated as

(1) $$c={\left(\frac{1}{c_{w}}-\frac{\Delta t}{l}\right)}^{-1}$$

where *c_w_* is the acoustic velocity in water and *l* the thickness of the gel sample.

During ultrasonic wave velocity measurements, tissue-mimicking phantoms doped with SNPs or CNPs were immersed in distilled water and placed between transmitter and receiver. For measurement of SNP and CNP suspension, the measuring chamber was filled with them. The resulting sound velocities *c* are presented in Table 1.

#### 2.3.2. Density Measurements

The phantom density was established according to Archimedes’ principle. The phantoms were first weighted in air and then weighted again after submersion in distilled water. Density was calculated using the formula $\rho =\frac{m_{a}}{\left(m_{a}-m_{aw}\right)}\rho_{w}$, where $m_{a}$ is the mass of the phantom in air, $m_{aw}$ is the mass of the phantom submerged in water, and $\rho_{w}$ is the density of distilled water. For precise weighting, analytical laboratory balances (Radwag AS 220/X; Radwag, Radom, Poland), with readability to 1 mg, were used.

#### 2.3.3. Hyperthermia Measurements

The heating power of magnetic nanomaterials can be calculated using calorimetric measurements. The specific absorption rate (SAR) volume was evaluated to characterize the power deposition in the tissue during the thermal treatment. The SAR value depends on the efficiency of the heat source and the ability of the heated medium to absorb thermal energy. Thus, in the case of magnetic particle heating, the SAR value depends on the concentration of nanoparticles and their ability to rotate (Brown mechanism) in dense material. According to the usual definition, the SAR describes the rate of energy absorption by a material and can be calculated as:

(2) $$\mathrm{SAR}=c_{p}{\left(\frac{dT}{dt}\right)}_{t=0}$$

where *c_p_* is the specific heat of the phantom. In practice, obtaining the SAR experimentally involves measurement of the temperature increase following a step-in heating, fitting the resulting data to a linear function in time, and then determining its slope at time zero.

**Figure 3:**
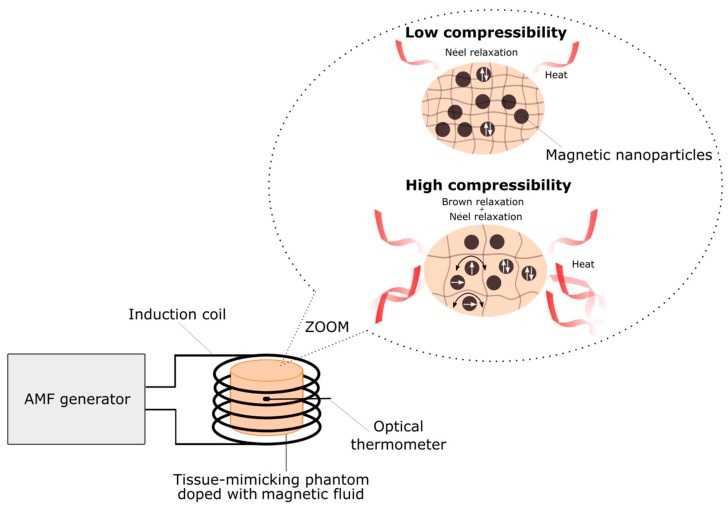
Experimental setup for an alternating magnetic field (AMF) hyperthermia measurements.

The compact EASYHEAT (Ambrell Corporation, Scottsville, NY, United States) induction heating system was used as a source for an alternating magnetic field. The induction heating setup consists of a high-frequency power supply that takes the input from the AC line mains. The high-oscillating signal is then fed to a tank circuit that feeds the water-cooled induction heating coil. The high-frequency electric signal generates a high-frequency magnetic field inside the induction coil. The phantom was inserted into a sample cell that was centrally placed and thermally insulated in a water-cooled magnetic induction coil. The frequency of the alternating magnetic field is 356 kHz and the maximal intensity is 16.2 kA/m. The temperature in the phantom during all hyperthermia experiments was measured using a FLUOTEMP temperature sensor system (Photon Control Inc., Burnaby, BC, Canada) with the optic fiber temperature probe (model FTP-NY2). The signal from the probe is unaffected by the magnetic field and other interferences. During experiments, the optical fiber was centrally placed in the phantom. The experimental setup for magnetic heating measurements is presented in Figure 3.

## 3. Results and Discussion

### 3.1. Elastic Properties

To conduct all experiments in conditions similar to human tissues of various mechanical properties, we prepared phantoms with different concentrations of agar *ϕ_a_*, resulting in various elastic properties. Based on the experimental results of sound velocity and density measurements, the bulk modulus and compressibility of SNP/CNP water suspension and agar phantoms doped with those magnetic fluids were calculated. The obtained values of sound velocity, density, bulk modulus and compressibility are presented in Table 1.

**Figure 4:**
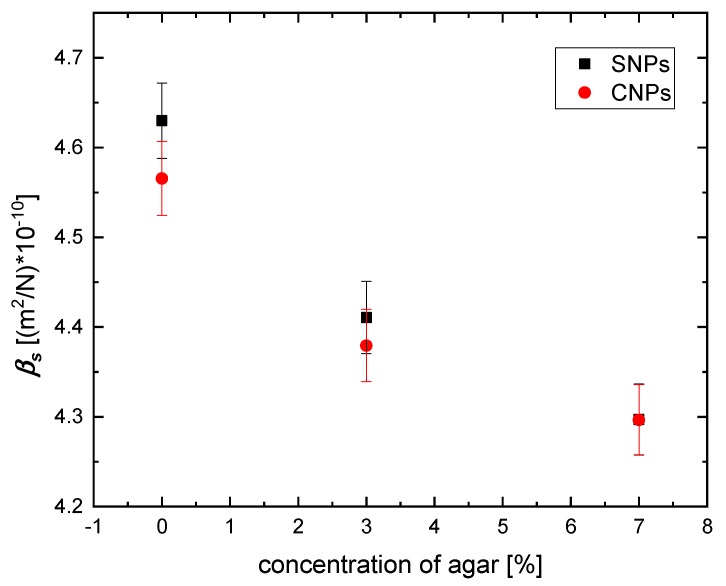
Compressibility of tissue-mimicking phantoms doped with SNPs or CNPs for different concentrations of agar.

Tissue-mimicking phantoms with a higher agar concentration *ϕ_a_* have a higher bulk modulus *K*, which noticeably corresponds to the stiffness of the prepared material. The stiffness of agar phantoms depends heavily on the concentration of agar; therefore, it is appropriate to study in them the effectiveness of hyperthermia as a function of phantom mechanical properties. It can also be seen that density and sound velocity increase with agar concentration *ϕ_a_* in tissue-mimicking phantoms, while the compressibility decreases. Moreover, the obtained compressibilities for tissue-mimicking phantoms doped with magnetic material are lower than the compressibilities for pure agar phantoms that can be found in part 2.2. Interestingly, as shown in Figure 4, with increasing agar concentration *ϕ_a_* in the phantom, the difference between used magnetic suspensions diminishes. It can be concluded that the stiffness of the material for the 7% agar phantom is so substantial that the characteristic of the used magnetic material (overall size and type: single, cluster) do not influence phantom compressibility.

### 3.2. Magnetic Hyperthermia

The heating effect of magnetic particles is a result of an absorbing energy from the alternating magnetic field and its conversion into heat. There are three mechanisms responsible for this effect: eddy current losses, hysteresis losses during reversal of magnetization, and relaxation losses accompanying demagnetization. However, to single domain nanoparticles, only the relaxation mechanism applies. 

Néel relaxation dominates for small particles (diameter up to 14 nm). Brown relaxation dominates for larger sizes (diameter above 14 nm) [24]. The distribution curve of nanoparticle size is not narrow. Therefore, it can be concluded that both mechanisms of relaxation will be present during magnetic hyperthermia. As the Brown and Néel processes contributes to the overall heating, the effective relaxation time is given by

(3) $$\tau_{eff}=\frac{\tau_{B}\cdot \tau_{N}}{\tau_{B}+\tau_{N}}$$

where $\tau_{N}$ is Néel relaxation time and $\tau_{B}$ is Brown relaxation time. The Néel relaxation, which does not depend on viscoelastic properties, can be described as

(4) $$\tau_{N}=\tau_{o}exp\left(\frac{KV}{k_{B}T}\right)$$

where *KV* is the magnetic anisotropy energy barrier, $k_{B}$ is the Boltzmann constant, and T is the temperature.

The Brown relaxation mechanism originates from the rotation of the whole particle in a viscous carrier fluid and is described by a relaxation time $\tau_{B}$, which depends on the hydrodynamic volume *V*_h_, shear viscosity *η*_s_, and temperature *T*

(5) $$\tau_{B}=\frac{3\eta_{s}\cdot V_{h}}{k_{B}T}.$$

In order to set the magnetic moment in the direction of the applied magnetic field, the whole magnetic particle must rotate. The Brown effect in dense materials, such as gels or tissues, is very limited due to the fairly fixed position the nanoparticles occupy in the medium. In magnetic fluids, nanoparticles move freely and generate heat easily. To confirm this assumption, magnetic heating experiments as a function of phantom compressibility were conducted.

**Figure 5:**
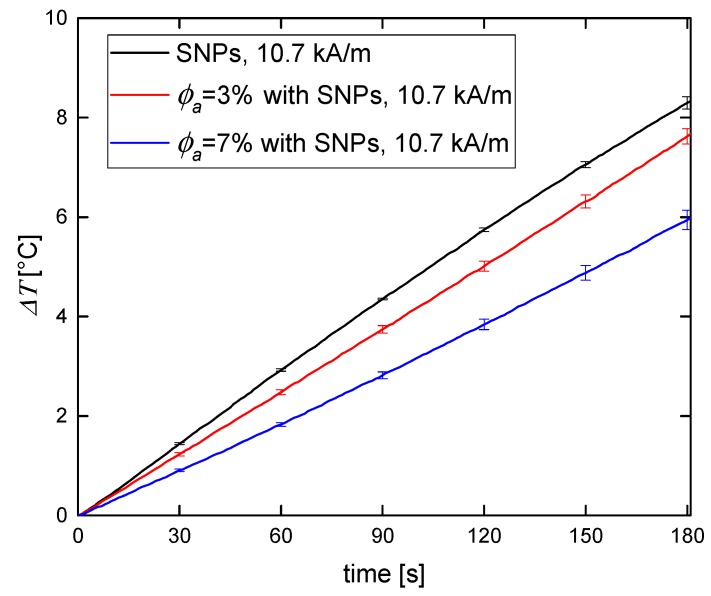
Temperature increase for SNPs suspended in water and embedded in 3% and 7% agar phantom for 10.7 kA/m of the magnetic field.

**Figure 6:**
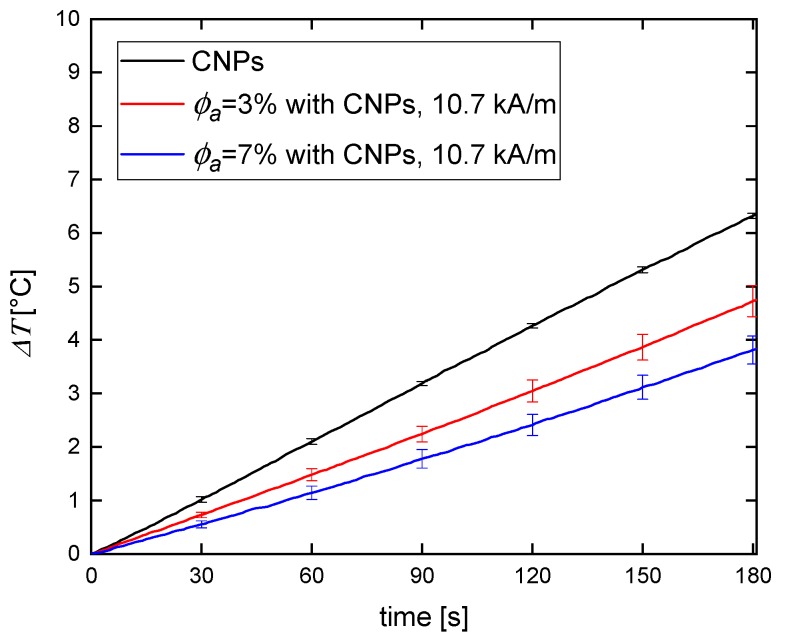
Temperature increase for CNPs suspended in water and embedded in 3% and 7% agar phantom for 10.7 kA/m of the magnetic field.

The results of magnetic heating performed on phantoms were compared to the results performed on SNP and CNP water suspensions (Figure 5 and Figure 6).

Regardless of the type of the magnetic material used in the phantom, the temperature increase for 3% agar phantom was higher than for 7% agar phantom. In Table 1 we showed that the higher the agar concentration of the phantom, the lower its compressibility. Thus, the observed decrease in magnetic heating efficiency is a consequence of the inhibition of Brown relaxation (physical particle rotation). Néel relaxation (magnetic moment rotation) is independent of the stiffness of the medium in which magnetic particles are embedded, so it does not vary from concentration to concentration. The differences in hyperthermia efficiency between water suspensions of magnetic nanoparticles and tissue-mimicking phantoms were recently reported by Avolio et al. [25]. They proved that when using smaller magnetic nanoparticles (around 10 nm in size), the obtained efficiency (expressed by the SAR value) was comparable for both water suspension and hydrogel doped with nanoparticles. As mentioned above, it is the diameter’s range of Néel relaxation domination. Otherwise, for bigger nanoparticles from the range of Brown relaxation, the observable difference between water suspensions and phantoms was indicated.

**Figure 7:**
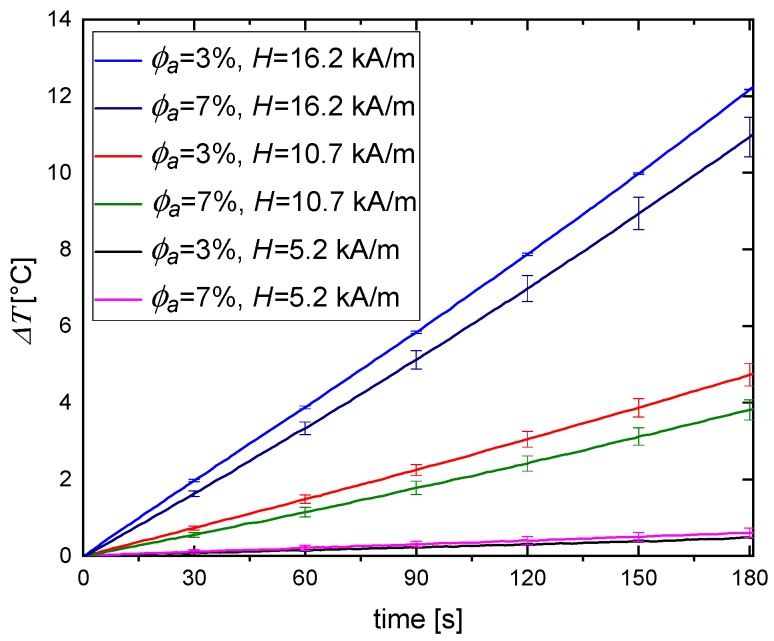
Temperature increase for CNPs embedded in 3% and 7% agar phantom during the heating in AMF at 5.2, 10.7, and 16.2 kA/m strength.

Our magnetic hyperthermia experiments for CNPs embedded in agar phantoms with various values of stiffness were also performed for several values of magnetic fields: 5.2, 10.7, and 16.2 kA/m. Figure 7 shows that the higher the magnetic field, the higher the temperature increase. These results prove again that agar concentration, used to form tissue-mimicking phantoms, has an impact on the obtained temperature rise. For each experimental line in Figure 7, one can see the differences between 3% and 7% agar concentrations.

**Figure 8:**
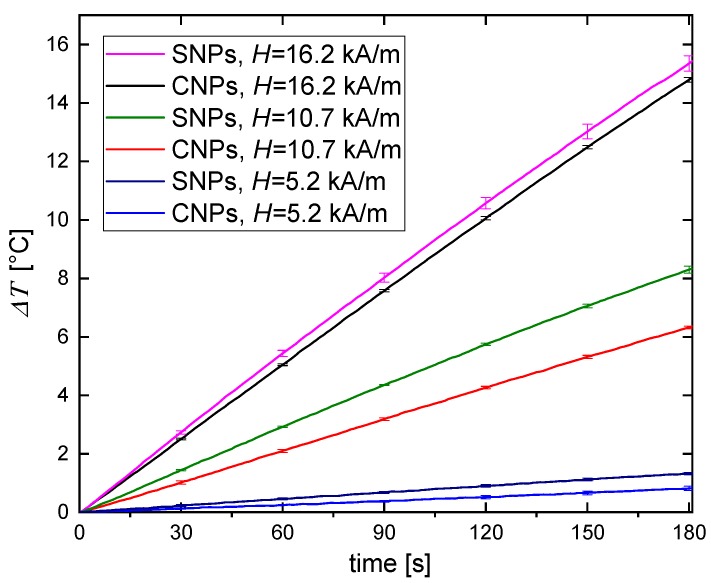
Comparison of temperature increase for SNP and CNP water suspension during heating in AMF at 5.2, 10.7 and 16.2 kA/m strength.

For our experiments, we used two types of magnetic particles: single and cluster nanoparticles. Although the size of a single nanoparticle separated from CNPs has a similar size to SNPs, the overall size of the CNPs is larger, as shown in Figure 1a,b. For that reason, CNPs in an alternating magnetic field move less freely than SNPs, which results in less effective heating than SNPs, regardless of the magnetic field intensity. This trend is especially visible when combining the results for temperature rises in the same magnetic field conditions for both single and cluster nanoparticles (Figure 8). The above conclusions correspond well with the other works on magnetic heating efficiency in hydrogels. Engelmann et al. [26] recently indicated that the macroscopic structure of the gel, in which magnetic nanoparticles are embedded (mesh size), can hinder Brown relaxation for sufficiently large magnetic objects. This effect is not the only one that explains the lower efficiency of magnetic hyperthermia for cluster nanoparticles. It has been reported that the dipole interactions of magnetic nanoparticles in a cluster can also influence heating. When the clusters are isotropic in shape, heating efficiency is lower than that of non-interacting particles despite the cluster’s size [27]. Other authors [28] reported that heating efficiency for maghemite clusters (45–98 nm) in water media is better than for single nanoparticles (13 nm), due to the fact that the surface of SNPs cools down more rapidly than the surface of CNPs. However, as the authors also indicated, the heating efficiency of clusters decreases with the increase of their size. The highest heating efficiency is for the cluster size around 50 nm, but for the bigger cluster, it clearly starts to decrease. It can be assumed that with a further decrease of CNP size, the same, or even worse, heating effects observed for SNPs can be expected for CNPs. In our work, we used magnetite CNPs with sizes much larger than 50 nm (110–130 nm), so we obtained a worse heating effect for clusters than for single nanoparticles. The mentioned phenomena may also play a role, and act collectively, in our experiments. 

**Figure 9:**
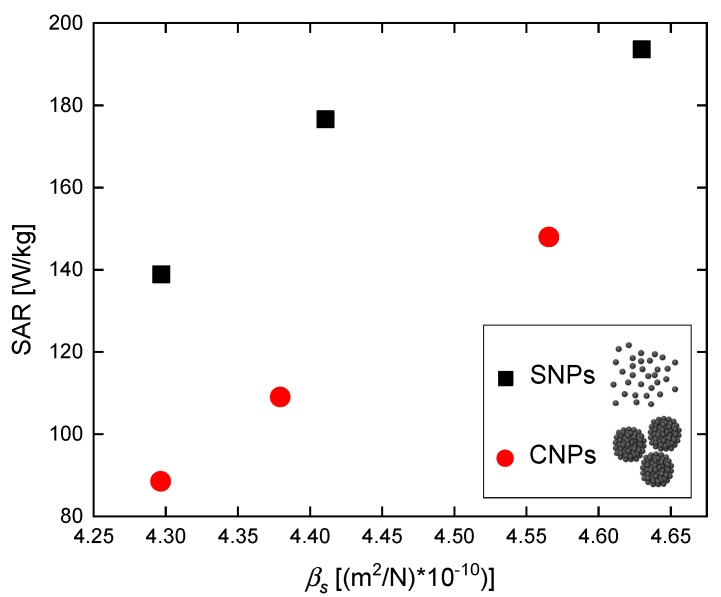
Specific absorption rate in a function of compressibility of tissue-mimicking phantom doped with SNPs or CNPs.

To characterize the power dissipation in the phantom, during the magnetic hyperthermia experiments, the SAR was evaluated. The SAR value provides information about the efficiency of the heat source (rate of temperature rise) and the ability of the heated medium to absorb thermal energy—see Equation (2). As we proved above, it depends on the nanoparticles’ ability to rotate (Brown mechanism). Therefore, we calculated the SAR as a function of phantom compressibility *β_s_*. The results are shown in Figure 9.

Our calculations show that the SAR values increase with increasing compressibility *β_s_*. This result again proves that tissue-mimicking phantom compressibility affects the effectiveness of the magnetic hyperthermia effect.

The low thermal effect of magnetic hyperthermia obtained in tissues can be improved. Combining magnetic hyperthermia treatment with other therapeutic methods, such as ultrasonic heating, can lead to a more effective output. In our previous work concerning magneto-ultrasonic heating [29], we demonstrated that a bimodal stimulation of nanoparticles provides better heating efficiency. Moreover, additional heating caused by ultrasound sonication may increase the phantom pores (as in sonophoresis treatment), allowing nanoparticles to move more freely. In this case, magnetic hyperthermia is improved by unlocking the Brown mechanism. 

## 4. Conclusions

Our research showed that in fluid suspensions, magnetic particles move freely and generate heat easily. In tissue-mimicking phantoms, whose mechanical properties are different, nanoparticle movement is limited, and the generated heat is smaller. The firm gel structure of the phantom does not affect the Néel mechanism. However, the higher the agar concentration of the phantom, the lower the compressibility, which leads to an inhibition of Brown relaxation, resulting in less effective magnetic heating. This is independent of the form of the nanoparticles (single vs. cluster). When medical usage of magnetic hyperthermia is considered, deterioration of the heating efficiency of the magnetic fluid, after being introduced into the body, should be considered. Nowadays, awareness of this fact in scientific reports is increasing [25]. Despite the low thermal effect of magnetic hyperthermia, its effectiveness can be improved. Combining magnetic hyperthermia treatment with other therapeutic methods, such as ultrasonic heating, can lead to more effective heating due to the unblocking of the Brown thermal mechanism [29]. Our research also showed that for low compressibility materials (phantoms with high agar concentration), there is no difference in heating efficiency for both types of magnetic materials (single or cluster nanoparticles). However, for materials with higher compressibility (phantoms with low agar concentration), the matter is different. The heating efficiency of single nanoparticles is higher than that of cluster nanoparticles. 

## Figures and Tables

**Table 1:** Sound velocity, density, bulk modulus and compressibility of agar phantom doped with SNPs/CNPs with different *ϕ_a_* agar concentrations.

Agar Concentration $\phi_{a}$ [%]	Type of Nanomaterials	Sound Velocity [m/s]	Density [kg/m^3^]	Bulk Modulus [GPa]	Compressibility β_s_ [(m^2^/N)]
0	SNPs	1479.29	987	2.160	4.630 × 10^−10^
3	SNPs	1498.30	1010	2.267	4.411 × 10^−10^
7	SNPs	1509.00	1022	2.327	4.297 × 10^−10^
0	CNPs	1483.68	995	2.190	4.566 × 10^−10^
3	CNPs	1503.60	1010	2.283	4.379 × 10^−10^
7	CNPs	1507.60	1024	2.328	4.297 × 10^−10^
